# Development of an Iberian Chorizo Salted With a Combination of Mineral Salts (Seawater Substitute) and Better Nutritional Profile

**DOI:** 10.3389/fnut.2021.642726

**Published:** 2021-03-04

**Authors:** Luis Tejada, Laura Buendía-Moreno, Elisa Álvarez, Agustín Palma, Eva Salazar, Beatriz Muñoz, Adela Abellán

**Affiliations:** ^1^Department of Human Nutrition and Food Technology, Universidad Católica de Murcia-UCAM, Murcia, Spain; ^2^Agencia Española de Seguridad Alimentaria y Nutrición, Madrid, Spain

**Keywords:** sodium chloride replacement, nutrition properties, nutrition and health food labeling, sausage, mineral salt (Winbi)

## Abstract

The present study evaluated the effect of salt reduction using a seawater substitutes, at the nutritional and mineral composition, its physicochemical, biochemical, microbiological, and sensory characteristics of Iberian chorizo, compared with one elaborated with low salt content (KCl) and another with a normal salt content (CTRL). To this end, three batches of chorizo were prepared [Treatment 1: CTRL, 100% NaCl; Treatment 2: KCl, 31% KCl, and Treatment 3: SC (Winbi®), <3% NaCl]. In KCl and SC chorizo lots, values of moisture, salt, and water activity (a_w_) were significantly lower (*P* < 0.05) than in the CTRL chorizo. The chorizo with lower salt content presented higher proteolytic activity; with the nutritional declaration “reduced Na content “with Na values 25% lower than the CTRL. In addition, using this combination caused significant effects (*P* < 0.05) on the mineral composition of chorizo SC, allowing the inclusion of more nutritional and health claims in its labeling under legislation. The partial substitution of NaCl for KCl (31%), caused an increase in the gumminess, chewiness, and hardness of the chorizo. The SC chorizo lost the reddish hue typical of this sausage, although it was the best sensory valued by a panel of consumers. No differences were observed in the microbiological quality of the different batches of chorizo, always fulfilling the legally established microbiological criteria.

## Introduction

Salt has been a preservative in food for thousands of years. The discovery of other preservation methods, such as refrigeration, has allowed the reduction of this food additive, but it is still not enough (1). According to the World Health Organization, high blood pressure and cardiovascular diseases are the leading cause of death worldwide (2). One of the primary determinants involved in the origin of high blood pressure is the excessive consumption of sodium that is ingested in the diet as sodium chloride (common salt; NaCl), its intake linked to kidney diseases and increases in blood pressure (3). In Europe, the average daily consumption of common salt is estimated to be 8.11 g/day, well above the 5 g/day recommended (4).

Currently, the reformulation of food to improve its healthiness is an important aspect and a challenge for the food industry. Therefore, the framework of the European Green Pact and the strategy “from farm to fork,” propose actions that, synergistically, help consumers in choosing healthy diets and sustainable. These measures comprise the establishment of nutritional profiles (with thresholds for nutrients such as fats, sugars, and salt) that regulate the use of nutritional and health claims contemplated in Regulation (EC) No. 1924/2006, as well as nutritional labeling on the front-of-package that makes it easier for consumers to understand the nutritional composition of foods.

Salt is a component widely used in the food industry, as it can improve flavors, prevent microbial growth and enzymatic activity, as well as guarantee a characteristic texture and flavor in the food (5, 6). In Europe, around 75% of the salt consumed comes from processed foods, of which 20% is derived from meat products (7). Spain has one of the highest consumption and production rates of meat products, which justifies the need to substitute or reduce the salt content in this food.

Raw cured sausages are meat derivatives in which their consumer acceptability is mainly due to the fundamental role that salt has on their microbiological, physicochemical, and sensory quality (8). Using NaCl to produce sausages is of vital importance, since it reduces the water activity of the food, thus preventing the development of microorganisms capable of altering the quality of the final product (9). In addition, NaCl has a protein solubilisation effect due to the increase in ionic strength and its subsequent gelation and binding of the particles that make up the mass, until reaching a suitable consistency and texture (10). Salt also conditions the biochemical and enzymatic reactions that take place during the maturation of cured raw sausages, affecting the aroma of the product, besides partially fragmenting the proteins that lead to the release of non-protein nitrogenous compounds, affecting the pH, flavor, and aroma of the sausage (11).

Given the functions that NaCl performs on cured raw sausages, reducing its concentration in this meat product is especially difficult. An alternative to salt reduction is the partial substitution of NaCl with other salts or ingredients (KCl, CaCl_2_, MgCl_2_, K-lactate, and glycine, among others) that provide the same technological functions, while maintaining their sensory characteristics, obtaining products that represent an improvement in their nutritional properties (12). The substitutions of NaCl using potassium salts are the most implemented in the reduction of Na. Some researchers studied the effect of reducing NaCl, up to 50%, by using K salts, without producing unfavorable changes in the sensory and microbiological characteristics in sausages (13). Using KCl has been limited mainly by its bitter-metallic taste, but studies have shown that the combination of KCl with NaCl of up to 50% does not detect variations in taste and texture of cooked ham (14), nor in sausages and pork loin (15), agreeing with other studies (16). Some authors confirm that the partial substitution of NaCl by KCl seems to be the best alternative to reduce the sodium content in meat products, since both salts have similar properties and the consumption of K has not been related to developing high blood pressure and cardiovascular diseases (17). Therefore, the partial substitution of NaCl by KCl has been the most common strategy to reduce the salt content in manufactured meat derivatives, but usually with unsatisfactory results. There is also no evidence of the partial substitution of NaCl by KCl in the production of Iberian chorizo, a raw meat derivative cured with high consumption in Spain.

Sea salt an alternative used in the culinary preparation of some foods, as a substitute for NaCl, providing sensory characteristics highly appreciated by the consumer, with greater amounts of minerals and trace elements than NaCl (18). Recently, seawater substitutes with a similar composition have been developed, which solve the inconveniences of treating and transporting seawater. No evidence shows using a combination of salts (seawater substitute) to replace NaCl in the production of cured sausages, nor have studies investigated the reduction of NaCl in Iberian chorizo using KCl as an alternative. This combination of salts, besides reducing the Na content and providing genuine sensory characteristics, increases the concentration of other minerals, allowing the inclusion of some nutritional and health claims in food on its labeling, which are included in the Regulation (CE) 1924/2006 of the European Parliament and of the Council, as “reduced sodium/salt content.”

The objective of this study is to develop an Iberian chorizo with better nutritional properties (reduced Na content and greater contribution of other minerals), maintaining or even improving its hygienic quality, sanitary, and organoleptic properties, using a combination of mineral salts, compared to other chorizo produced with KCl and NaCl.

## Materials and Methods

### Samples

Three batches of 12 Iberian chorizos were made in the pilot plant of a Spanish meat company (Treatment 1: CTRL, four samples; Treatment 2: KCl, four samples and Treatment 3: SC, four samples). Table 1 includes the ingredients used for each formulation (some of them confidential by the meat company). Treatment 1 is the control formulation, with 100% NaCl; Treatment 2, the KCl formulation has 31% of the NaCl replaced by KCl (31% KCl + 69% NaCl); also, a masking aroma was incorporated to avoid possible bitter flavors derived from KCl; furthermore Treatment 3, the SC formulation, where 100% NaCl has been replaced with 97% of combined mineral salts (97% SC + 3% NaCl) (Winbi® whose Na content is 25% lower than the control). The composition of the combined mineral salts (Winbi®) is shown in Table 2. As a starter, a mixture of *Staphylococcus xylosus* and *Pediococcus pentosaceus*, supplied by the Oleica company (Guaro, Malaga, Spain) was used. The manufacturing process was the same for all batches. First, the meat and fat were minced in a mincer with 60 and 10 mm orifice plates. Second, the rest of the ingredients were incorporated into the mass, mixing them using a vacuum mixer for approximately 4 min. Subsequently, each mass was stuffed into collagen casings of caliber 47, forming pieces with an average weight of 695 g. After draining the pieces, they were transferred to a dryer with a temperature of 5 ± 2°C and 70–85% of relative humidity (RH), where they remained for 21 days. They were then moved to a cellar with a controlled temperature between 12 and 5°C and RH around 70–85% for 27 days. Finally, the chorizos were stored in a natural cellar until the end of their curing, 30 days later, for which the total maturation time was 57 days. To conduct the analyses, first, aseptically, the sample was taken for microbiological analysis. Subsequently, the rest of the chorizo was crushed, where a first part was used to determine moisture, pH, and a_w_, the remaining was frozen until the moment of use.

**Table 1:** Composition of the different formulations used in the production of Iberian chorizo, expressed as g/Kg of meat.

**Ingredients**	**CTRL**	**KCl**	**SC**
Paprika	14.31	14.31	14.31
Garlic paste	1.67	1.67	1.67
Oregano	0.14	0.14	0.14
Additive^a^	9.54	9.54	9.54
Nitrifying salt	1.43	1.43	1.43
NaCl	19.08	7.15	-
Salts combined	-	-	18.49
NaCl + KCl + Aroma	-	11.92	-

a *Monosodium glutamate and aroma masker for taste*.

**Table 2:** Composition of salts combined Winbi®.

**Ingredients**	**Minerals**	**100 g**	**By unit (1.2 g)**
Salt	-	73.7 g	0.9 g
Sodium chloride	Ion Sodium (Na^+^)	29.49 g	0.35 g
Magnesium sulfate	Ion Magnesium (Mg ^2+^)	2,976 mg	35.71 mg
Magnesium chloride	Ion Chloride (Cl-)	50,849 mg	610.19 mg
Natural sodium sulfate	Ion Sulfate (SO${}_{4}^{2-}$)	7,891 mg	94.69 mg
Calcium chloride	Ion Calcium (Ca^2+^)	1,091 mg	13.09 mg
Potassium chloride	Ion Potassium (K^+^)	1,364 mg	16.37 mg
Sodium bicarbonate	Ion Bicarbonate (HCO^3−^)	1,017 mg	12.20 mg

### Physicochemical Analysis, Proteolysis, and Mineral Composition

Weight loss was determined by weighing each sample, in triplicate, throughout the entire curing period (80 days). The results were expressed as a percentage of weight loss regarding the initial weight of the whole product.

The moisture, protein, fat, and ash content were determined according to the methods 950.468, 981.10, 920.153, and 960.39(b), respectively, established by the AOAC. All tests were performed in triplicate. The sodium content was determined in triplicate for each formulation using an inductively coupled plasma atomic emission spectrophotometer (ICP-AES Ultima 2, Horiba Jobin Yvon, Milan, Italy). The amount of salt was estimated considering all the Na was in NaCl. The pH was measured using a HI 99163 pH meter (Hanna Instruments Inc., Hoonsocket, Dakota del Sur, USA) as established in the ISO 2917:1999 standard (19). For this, 10 g of each sample with distilled water in a 1:10 ratio in triplicate. The a_w_ measurement was performed, in triplicate, with a Novasina meter (LabSwift, Metrohm AG, Madrid, Spain) following the AOAC 978.18 method (20).

Non-protein nitrogen (NPN) was determined using the Kjeldahl method, after protein precipitation, using 12.5% trichloroacetic acid (TCA) (21). The results were expressed as mean values in g of nitrogen/kg of dry matter. The proteolysis index (PI) was calculated as the percentage ratio between NPN and total nitrogen (TN) (22).

Mineral determination was conducted using an ICP-AES Ultima 2 (Horiba Jobin Yvon, Milan, Italy). For this, 0.5 g of crushed Iberian chorizo was mineralised in a microwave system (Milestone 1200, FVK, Bergamo, Italy) using 3 mL of nitric acid (Romil Ltd., Cambridge, United Kingdom) and 0.5 mL of Suprapur 30% hydrogen peroxide (Merck, Germany). The samples were diluted with 50 mL of milliQ water and read at different wavelengths: Na (589.59 nm), K (766.49 nm), Ca (393.36 nm), Mg (279.55 nm), P (213.61 nm), Fe (238.20 nm), Cu (346 nm), Mn (540 nm), Zn (213.85 nm), and B (532 nm) (23). The content of each mineral was expressed in mg/100 g of meat.

### Instrumental Analysis

#### Color Measurement

The color was determined using a Color Flex spectrocolorimeter [Hunter Associates Laboratory Inc., Reston, VA, USA (illuminant D65 and a visual angle of 10°)]. The established color parameters were: L^*^, a^*^, b^*^, C^*^, and H^*^. Three readings were made per formulation in different slices of the Iberian chorizo at the end of the curing (24).

#### Texture Profile Analysis (TPA)

Three meat pieces of 2.5 cm of thickness were cut and were subjected to a texture analysis using a Texture Analyzer (TAXT2, Stable Micro System, UK), at 25°C and under the following study conditions: an activation load of 0.44 N, pre-test speed of 2 mm/s, post-test speed of 5 mm/s, distance of 8 mm and a force of 5 g. The texture parameters obtain were hardness, adhesiveness, cohesiveness, elasticity, gumminess, and chewiness.

### Sensory Analysis

The study was conducted in a standard room equipped with individual cabins according to this method (25). The panel of tasters comprised 60 untrained people who were given a questionnaire, along with the sliced samples to be tested, which were coded with three random numbers. The sliced samples were served at 25°C and arranged on plates. An acceptance test was performed using a 5-point hedonic scale, considering the rating of 5 (I like it very much) and 1 (I dislike it very much). The sensory attributes established for this study were: color, odor, salty taste, texture, and global assessment. The ethics committee of the Universidad Católica San Antonio does not believe that a declaration of ethics is appropriate for the development of the sensory analysis of this product, as it complied with all food safety requirements.

### Microbiological Analysis

Twenty-five grams of sausage sample were homogenized with 225 mL of peptone water (Scharlau Chemie S.A., Barcelona, Spain) in a Stomacher LabBlender 400 (Seward Medical, London, UK) for 1 min. Dilutions were made from the resulting suspension using peptone water. The quantification of *Salmonella* spp and *Shigella* bacteria were carried out by sowing 0.1 mL of each dilution on XLD agar (Scharlau Chemie S.A., Barcelona, Spain), and *Listeria monocytogenes* was sowed on Oxford agar (Scharlau Chemie S.A., Barcelona, Spain). For the differentiation of species of the genus *Staphylococcus*, 1 mL of the dilutions was added in Baird Parker Agar (Charlau Chemie S.A., Barcelona, Spain). Each dilution was seeded in triplicate for both media and the plates were incubated at 30°C/24 h, for *L. monocytogenes*, and at 37°C/24 h for *Salmonella spp, Shigella*, and *Staphylococcus aureus*. The results were expressed as colony-forming units per gram of Iberian chorizo (CFU/g).

### Statistical Analysis

The determinations, except for the sensory analysis, were conducted in triplicate and the results were expressed as the mean and standard deviation. For the sensory analysis only one sample was used for each formulation, so the final scores were averaged over all the panelists. Analysis of variance using a one-way ANOVA procedure and Tukey's test were performed to determine the effect of the different formulations (CTRL, sodium chloride; KCl, potassium chloride; and SC, combined salts) on all the parameters studied in Iberian chorizo at the end of the curing period. The statistical differences were given when *P* < 0.05. Statistical analysis was conducted using the SPSS version 21.0 software package (IBM Corporation, Armonk, NY, USA).

## Results and Discussion

### Physicochemical Analysis, Proteolysis, and Mineral Composition of Iberian Chorizo at the End of Its Curing

Table 3 shows the results obtained from the nutritional composition, physicochemical parameters, and proteolysis of the three batches of Iberian chorizo (CTRL, KCl, and SC). The moisture content of cured meat products is important as it influences their stability and safety. Slight changes in moisture can change the texture and acceptability of the product (26). The chorizos made with the combined salts (SC) or with the partial substitution of NaCl for KCl (KCl), presented lower moisture values than those made with common salt (CTRL). These same results were obtained in an investigation whose objective was to replace NaCl with other salts (replacement, 1.9%), including KCl, to prepare salamis, where the moisture content was higher in salamis elaborated with NaCl than those with KCl (27). These moisture differences may be because the mixtures of salts, mainly formed by KCl, penetrate the meat easier, thus hindering the exit of water from inside the meat (28). This variation could also be justified based on the different a_w_ values presented by NaCl and KCl, since the a_w_ of a saturated solution of NaCl at 25°C is 0.753 and that of KCl is 0.843. Thus, when the air from the dryers maintains the a_w_ of the sausage surface between 0.753 and 0.843, the samples formed only by KCl will have a lower water content on their surface than the samples made with NaCl. With a lower water content on the food surface, the weight loss will be greater and the moisture content lower (29). The greater loss of water in SC and KCl is corroborated with the product weight loss during processing (Table 4). Significant differences were observed in weight loss between the different formulations from the 15th day of curing, observing higher values in KCl (17.99%) and SC (17.01%) sausages, regarding CTRL (14.88%). It was observed that the decrease during the entire ripening process was lower in chorizo made with NaCl (CTRL), with values of 34.12, 40.02, and 38.21% for the CTRL, KCl (formulation after 80 days), and SC, respectively.

**Table 3:** Nutrition composition, physicochemical parameters, and proteolysis measure of Iberian chorizo at the end of the curing processing.

**Nutrition Composition^**1**^**	**CTRL**	**KCl**	**SC**	***P*-value**
Moisture	19.42^b^±0.32	16.81^a^±0.39	17.42^a^±0.40	0.000344
Protein	28.68^a^±1.13	31.84^a^±0.53	31.96^a^±3.77	0.220577
Fat	35.98^a^±3.80	34.40^a^±2.31	32.03^a^±3.01	0.355542
Ash	6.71^a^±0.32	6.82^a^±0.05	6.58^a^±0.45	0.671721
Salt	3.24^b^±0.04	2.41^a^±0.24	2.36^a^±0.01	0.000460
**Physicochemical parameters**^**2**^				
pH	5.25^a^±0.02	5.4^a^±0.15	5.2^a^±0.10	0.053
a_w_	0.881^b^±0.003	0.875^a^±0.002	0.873^a^±0.002	0.0041
**Proteolysis**				
Non-protein nitrogen (g nitrogen/dry extract)	0.08^b^±0.01	0.14^a^±0.01	0.14^a^±0.00	0.005578
Proteolysis index	1.69^a^±0.01	2.73^b^±0.01	2.64^b^±0.02	0.0081

*Values are mean ± SD (n = 3). One-way ANOVA. Different letters in the same row indicate a significant different (P < 0.05) between the samples SC (salt combination), KCl (potassium chloride), and NaCl (sodium chloride, common salt)*.

*Treatment I: CTRL (100% NaCl); Treatment II: KCl (31% NaCl + 69% KCl); Treatment III: SC (3% NaCl + 97% SC)*.

*^1^g/100 g of Iberian chorizo*.

*^2^dimensionless*.

*a_w_, water activity*.

**Table 4:** Weight loss of Iberian chorizo during the curing processing.

**Days**	**CTRL**	**KCl**	**SC**	***P*-value**
0	0	0	0	
5	7.50^a^±1.11	8.08^a^±1.11	8.10^a^±0.88	0.81
10	12.10^a^±1.01	14.05^a^±1.66	13.77^a^±1.80	0.0650
15	14.88^b^±2.55	17.99^a^±1.22	17.01^a^±2.01	0.0475
25	17.44^b^±1.88	23.22^a^±3.40	22.47^a^±2.40	0.0423
30	21.02^b^±1.51	26.02^a^±2.80	24.98^a^±3.40	0.0404
35	23.44^c^±2.22	28.44^a^±3.55	26.80^b^±2.11	0.0338
45	25.45^c^±1.22	31.01^a^±2.01	28.23^b^±1.25	0.0465
50	27.12^c^±2.01	34.20^a^±2.11	31.54^b^±2.41	0.0275
60	30.02^c^±1.22	36.44^a^±1.88	34.24^b^±0.88	0.0350
70	33.45^b^±1.15	39.14^a^±0.88	37.44^a^±2.22	0.0175
80	34.12^b^±1.15	40.02^a^±0.80	38.21^a^±0.58	0.0458

*Values are given as mean ± SD (n = 3). One-way ANOVA. Different letters in the same row indicate a significant different (P < 0.05) between the samples SC (salt combination), KCl (potassium chloride) and NaCl (sodium chloride, common salt)*.

*Treatment I: CTRL (100% NaCl); Treatment II: KCl (31% NaCl + 69% KCl); Treatment III: SC (3% NaCl + 97% SC)*.

*The results are expressed as percentage weight loss (%)*.

No significant differences were observed in the content of protein, fat, and ash (*P* > 0.05). Nutritionally, in relation to proteins, it is shown that, for the three batches of chorizo, the nutritional declaration “high protein content” could be made, since proteins provide over 20% of the energy value of the food in these cases (30). In addition, CTRL, KCl, and SC can make the approved health claims that protein contributes to building muscle mass, maintaining muscle mass, and maintaining normal bones (i.e., bone calcium levels and bone density) (31).

The NaCl concentration was higher in CTRL (3.24/100 g) than in KCl and SC. Thus, the chorizo made with the combination of mineral salts (SC) obtained the greatest reduction in NaCl (2.36/100 g), followed by KCl (2.41/100 g). These results coincide with those obtained by Horita et al. who achieved a similar compatibility relationship by replacing NaCl with 50 and 75% KCl to prepare “mortadela” (a dry-cured luncheon meat) (32). Ibáñez et al. achieved Na reductions similar to those obtained in this study, by partially replacing NaCl with KCl in fermented sausages (33).

The pH and a_w_ are essential factors that guarantee the stability and safety of the sausages. Regarding pH, no significant differences (*P* > 0.05) were observed between the three formulations used to prepare Iberian chorizos (Table 3), agreeing with what was observed in manufacturing a typical Italian salami reduced in NaCl (23). Regarding a_w_, the values obtained in all chorizos were considered normal for these products, and adequately favor their storage and safety. In Table 3, slightly higher a_w_ values can be seen in the CTRL chorizo, probably due to their higher moisture content (19.42%) than the other formulations (KCl, 16.81% and SC, 17.42%). In addition, adding solutes, such as mineral salts, helps to reduce the value of a_w_, since when hydrated, the availability of water is reduced (34). Therefore, the chorizo made with the SC formulation has a lower a_w_ (0.873). Similar studies presented results approximate to those of this study, like Corral and Flores (6). These authors observed a decrease in a_w_ when replacing 50% of NaCl with KCl, besides concluding the need to search for other ingredients that, when combined with KCl, achieve greater substitution without causing alterations, especially sensory. However, another study conducted on fermented sausages shows that the highest a_w_ was developed in sausages made with the highest percentage of NaCl, as occurs in this study (6).

The NPN and the PI were lower in CTRL chorizos than in KCl and SC chorizos (Table 3). Some authors have observed that the activity of proteases (calpains and cathepsins) is diminished with the increase of the NaCl content, which justifies the lower PI in CTRL chorizos (21, 35, 36). NaCl has been shown to induce an inhibitory effect on the activity of cathepsins B and B + L. In cured loin some observed that the partial replacement of 50% of NaCl with KCl produces a higher activity of cathepsins B and B + L and, an increase in proteolysis, explaining this higher content of NPN in KCl and SC chorizos (17).

No references have been found on proteolysis in Iberian chorizo, and the values observed in other cured fermented products being highly variable, depending on the product and production process. Thus, in dry fermented sausages, NPN values of 8.8% were observed (37) and in dry sausage from Painho de Portalegre values were 0.85% (38). Furthermore, no studies have evaluated the effect of the substitution of NaCl on the NPN in cured raw sausages. In cured loin, some have also observed that with lower NaCl content the concentration of NPN was higher (21). In cured raw sausages only the effect of NaCl on free amino acids has been studied. Thus, an increase in the content of free amino acids (increased proteolysis) was developed in salamis in which NaCl was substituted with MgCl_2_ (27). Samples with a 50% reduction in NaCl showed a higher content of the amino acids Arg, Glu, His, Val, Cys, Lys, and Trp. Whereas, the samples formed by CaCl_2_ had a higher content of the amino acids Asp, Thr, Ala, Met, Leu, Ile, and Phe. The replacement of up to 50% of NaCl with KCl, in the salting stage to prepare cured loins, did not affect proteolysis (39).

The mineral content of the Iberian chorizo made with different formulations is shown in Table 5. The Na content decreased significantly with the reduction of the NaCl content, in the different formulations, up to 27.24% (KCl) and 25.71% (SC) compared to the CTRL. Therefore, according to Regulation (EC) 1924/2006, the nutritional declaration “reduced salt/sodium content” can be made on the labeling, presentation, and advertising of SC and KCl chorizos; complying with the conditions of use that the reduction of salt/sodium is at least 25%, compared to a similar product (CTRL). Simultaneously, it enables the SC and KCl chorizos to make the health claim “Reducing consumption of sodium contributes to the maintenance of normal blood pressure.”

**Table 5:** Mineral composition of Iberian chorizo at the end of curing processing.

	**CTRL^**1**^**	**KCl^**1**^**	**SC^**1**^**	***P-*value**	**RDA^**2**^**
Na	*1, 280*^b^±17.56	950^a^±96.25	930^a^±4.23	0.000000	0.006
K	620^a^±35.63	960^b^±16.64	640^a^±42.17	0.000024	2000
Ca	20^a^±1.41	30^a^±1.75	50^b^±7.73	0.001483	800
Mg	30^a^±1.00	40^a^±1.45	90^b^±1.78	0.000000	375
P	240^a^±12.73	280^a^±3.75	270^a^±19.33	0.064915	700
Fe	4.430^a^±0.14	3.810^a^±0.12	4.250^a^±0.14	0.805024	14
Cu	0.292^a^±0.00	0.332^a^±0.01	0.220^a^±0.00	0.091806	1
Mn	0.198^a^±0.00	0.095^a^±0.00	0.190^a^±0.00	0.550735	2
Zn	4.590^b^±0.01	5.550^a^±0.02	5.280^a^±0.04	0.014857	10
B	0.047^a^±0.00	0.043^a^±0.00	0.043^a^±0.00	0.932280	Not declared

*Values are mean ± SD (n = 3). One-way ANOVA. Different letters in the same row indicate a significant different (P < 0.05) between the samples: CTRL (sodium chloride, common salt), KCl (potassium chloride), and SC (salt combination)*.

*Na, sodium; K, potassium; Ca, calcium; Mg, magnesium; P, phosphorus; Fe, iron; Cu, copper; Mn, manganese; Zn, zinc; B, boron*.

*Treatment I: CTRL (100% NaCl); Treatment II: KCl (31% NaCl + 69% KCl); Treatment III: SC (3% NaCl + 97% SC)*.

*^1^Data are expressed in mg/100 g*.

*^2^RDA: Recommended Daily Allowances of the minerals*.

The K content of sausages made with the KCl formulation is higher (960 mg/100 g) than the CTRL (620 mg/100 g) and SC (640 mg/100 g), since the partial substitution of Na with K provides the food with a higher content of this mineral. Lorenzo et al. studied the elaboration of a cured pork shoulder using different salts as a replacement for NaCl, obtaining results like this study, as the partial replacement of Na with other salts (KCl 50% + NaCl 50%) reduced the Na content and increased K and Mg, compared to the control (100% NaCl) (40). Zanardi et al. obtained similar values in terms of K content. The salami formulated with a low concentration of NaCl (13.5 g/kg) presented a higher content of K (948 mg/100 g) than the control salami (530 mg/100 g) (23).

From a nutritional point of view, these results indicate that the CTRL chorizo contains 31% of the nutrient reference values (NRV) per 100 g, similar results are obtained for the K content in SC chorizo (32% of the NRV per 100 g). Whereas, the chorizo formulated with KCl contains 48% of the NRV per 100 g. Therefore, the CTRL, SC, and KCl chorizos can claim on their labeling the nutritional claims “source of potassium” and “high potassium” (41). In addition, the three samples analyzed as “source of potassium,” can make healthy statements on their labeling that “potassium contributes to normal functioning of the nervous system,” and “potassium contributes to the maintenance of normal blood pressure” (41). This last statement, for the SC and KCl Iberian chorizos, implies a possible synergistic action in relation to the cardiovascular system, together with the reduction in Na content. However, despite the K content in the KCl chorizo being significantly higher than in the CTRL and SC, for the purposes of statements there are no differences, and the consumer can verify this difference in content through the value reflected in the nutritional information of the product; where vitamins and minerals must be indicated in absolute amounts and %NRV for every 100 g or 100 mL of product.

Regarding Ca, the SC chorizos, present higher values (50 mg/100 g), producing an increase of 110 and 179% compared to KCl (30 mg/100 g) and CTRL (20 mg/100 g), respectively. The SC samples showed high Mg values (90 mg/100 g), compared to KCl (40 mg/100 g) and CTRL (30 mg/100 g). For both minerals (Ca and Mg) no significant differences were observed between the CTRL chorizos and those formulated with KCl. This may be due to the difficulty of divalent cations to penetrate inside the muscle (28). Another study conducted by Fieira et al. did not find significant differences in the content of minerals such as Ca and Mg in the elaboration of salami using different chloride salts (NaCl, KCl, CaCl_2_, and MgCl_2_) (42).

The NRV of Ca is 800 mg/100 g (41), thus the results obtained for Ca for the CTRL, KCl, and SC chorizos did not reach a significant amount (2.5, 3.75, and 6.25%, respectively); therefore, the nutrition claim “source of calcium” cannot be made, nor any health claims relating to this mineral. Regarding Mg, its NRV is 375 mg/100 g (41); here, results show that the CTRL chorizo contains 30 mg/100 g, the KCl chorizo contains 40 mg/100 g, and the SC chorizo contains 90 mg/100 g, as seen in Table 5; this represents 8, 10.66, and 24% of NRV, respectively. Therefore, only Iberian chorizo made with the SC formulation could make the nutritional claim “source of magnesium” on its labeling, and health claims authorized for this mineral. In relation to the products studied, it is worth highlighting the contribution of Mg to the electrolyte balance, performing an adjuvant action derived from the decrease in Na and increase in K in the formulation of SC and KCl chorizos.

The SC and KCl chorizos present higher *P*-values than the CTRL chorizo, but no significant differences were observed between the different formulations used (Table 5). Zanardi et al. did not observe significant differences of this mineral on the different formulations used in manufacturing an Italian-type salami (23). From a nutritional viewpoint, the NRV for P is 700 mg (41). According to the results obtained in this study, all chorizo samples could include in their labeling these nutritional declarations: “source of phosphorus” and “high phosphorus,” as for all formulations the calculation of the NRV is above that stipulated 15%, specifically: 34.28% for CTRL, 38.57% for SC, and 40% for KCl chorizos. Consequently, health claims authorized for P could also appear on its labeling (31).

In relation to the content of Fe, Cu, and Mn, in the Iberian chorizo samples, the results show similar figures without observing significant differences (*P* > 0.05) between the different formulations used. After performing the nutritional calculations based on the NRVs, the Iberian CTRL and SC chorizos can make the nutritional claims “source of iron” and “high iron,” while the chorizo made with the KCl formulation meets the conditions use only of the nutrition declaration “source of iron.” However, the three samples could make health claims authorized for the Fe (31). Nutritionally, the results for Cu represent a NRV of 33.2, 22.2, and 29.2% for the CTRL, SC, and KCl chorizos, respectively. According to these values, all samples can make the nutritional declaration of “source of cooper,” while the KCl formulation could also make the declaration of “high copper” on its labeling (41); thus authorized health claims can be made on all chorizo labeling (31). With Mn, in all three cases, there are discrete amount changes that do not reach significant values to make nutritional or health claims on their labeling.

With Zn, higher values are observed in the KCl (5.55 mg/100 g) and SC formulations (5.28 mg/100 g) compared to the CTRL (4.59 mg/100 g), which represents 55.5, 52.8, and 45.9% of the NRV (10 mg), respectively. Prepared salami, with different formulations, showed an increase in the Zn content, when the formulation comprised low concentrations of NaCl (23); corroborating results in this study. In all the samples the contribution of Zn is remarkable, which can be reflected in their labeling of the nutritional declarations “source of zinc” and “high zinc,” as well as the health claims authorized for this mineral (31). Of these statements, given the characteristics of the object of this study, it is worth highlighting those relating to “Zinc contributes to normal metabolism of fatty acids,” and “Zinc contributes to the protection of cells from oxidative stress.”

Finally, the results related to B show similar values, without observing significant differences between the different formulations (Table 5). There is no established NRV for this mineral, nor is there any authorized nutrition or health claim.

### Instrumental Analysis of the Iberian Chorizo at the End of Its Curing

Table 6 shows the mean values of the parameters of color, texture, and sensory analysis of the Iberian chorizo. Significant differences (*P* < 0.05) were observed in the color parameters L^*^, b^*^, C^*^, and H^*^ in the different formulations used. The CTRL chorizo showed a higher luminosity value (L^*^) (37.86) than the KCl (30.63) and SC (33.25) chorizos. Therefore, there has been a darkening of the SC and KCl chorizos, being more evident in the latter.

**Table 6:** Instrumental color measure, TPA, and sensory attributes and consumer acceptability of Iberian chorizo at the end of the curing processing.

**Color parameters**	**CTRL**	**KCl**	**SC**	***P*-value**
L*	37.86^c^±0.98	30.63^a^±0.85	33.25^b^±0.79	0.000166
a*	27.75^a^±1.79	26.93^a^±0.53	24.15^a^±2.74	0.131291
b*	22.09^a^±2.73	21.29^a^±0.21	15.88^b^±0.70	0.006826
C*	35.47^a^±3.10	34.33^a^±0.28	28.91^b^±2.66	0.030975
h	38.43^a^±1.61	38.34^a^±0.84	33.46^b^±1.96	0.011909
**Texture Profile Analysis**				
Hardness	35.55^a^±9.90	82.62^b^±8.88	48.37^a^±14.32	0.003733
Adhesiveness	0.60^a^±0.14	0.50^a^±0.14	0.60^a^±0.56	0.946910
Cohesiveness	0.38^ab^±0.007	0.40^b^±0.02	0.33^a^±0.01	0.403560
Elasticity	1.80^a^±0.08	1.83^a^±0.10	1.85^a^±0.12	0.900776
Gumminess	17.56^ab^±2.38	32.94^b^±5.11	15.59^a^±2.73	0.044105
Chewiness	18.66^a^±4.08	60.35^b^±12.79	29.44^ab^±3.39	0.040178
**Sensory Attributes**				
Appearance	3.46^a^±0.97	3.66^ab^±1.12	4.04^a^±1.08	0.023381
Odor	3.52^a^±1.05	3.78^a^±1.01	3.78^a^±1.11	0.370412
Texture	3.38^a^±1.06	3.34^a^±1.08	3.82^b^±1.00	0.043171
Salty Taste	3.47^a^±0.86	3.66^ab^±0.93	3.84^b^±0.89	0.135018
Global Taste	3.57^a^±0.95	3.67^a^±0.96	4.28^b^±0.92	0.000474
Consumer acceptability	3.59^a^±0.95	3.77^a^±0.88	4.22^b^±1.05	0.004801

*Values are mean ± SD (n = 3). One-way ANOVA. Different letters in the same row indicate a significant different (P < 0.05) between the samples*.

*CTRL (sodium chloride, common salt), KCl (potassium chloride) and SC (salt combination)*.

*Treatment I: CTRL (100% NaCl); Treatment II: KCl (31% NaCl + 69% KCl); Treatment III: SC (3% NaCl + 97% SC)*.

L*, lightness; a*, redness; b* yellowness; C*, saturation; h, tone.

*Hardness (N, Newton); Adhesiveness (mJ, millijoules); Elasticity (mm, millimeters); Gumminess (N); Chewiness (mJ)*.

This color change may be due to the greater loss of water from the product observed during the curing period (43). The chromatic coordinate a^*^ did not show significant differences (*P* > 0.05) between the different formulations used. However, the other color parameters (b^*^, C^*^, and H^*^) were lower in chorizos made with the combination of mineral salts (SC), compared to the CTRL and KCl chorizos. The few color differences between the CTRL, KCl, and SC chorizo, when reducing the NaCl content, is due to the chemical nature and ionic strength provided by the components used as substitutes (44). Although the reduction or replacement of NaCl by other salts develops a loss of the reddish hue typical of the pre-cured mixture. These results agree with those obtained from studying the influence of the replacement of NaCl by KCl to prepare a dry meat sausage (45).

Regarding the results obtained in the TPA, of the different chorizos analyzed (Table 6), adhesiveness and elasticity are the only parameters showing no significant differences were observed (*P* > 0.05) between the formulations. However, the KCl chorizo showed higher values of hardness, cohesiveness, gumminess, and chewiness, than the SC chorizo. The increased hardness of KCl chorizo may be related to the percentage of salt used during processing. Previous studies have shown that salt (NaCl) inhibits the activity of cathepsins B and B+L (17). However, the presence of high concentrations of KCl implies a higher activity of these cathepsins, and consequently an inhibition of proteolysis is produced (17). Therefore, in this study, the KCl chorizo presented higher hardness values (82.62 N) than the CTRL (35.55 N) and SC (48.37 N) chorizos. Corral and Flores also observed a decrease in cohesiveness and chewiness in the elaboration of sausage, when the NaCl content was reduced (46). Laranjo et al. also observed significant differences in cohesiveness with samples made with the highest percentage of salt presenting the highest values (47). However, Cittadini et al. found no significant differences in the cohesiveness, gumminess, and chewiness of jerky samples made with different percentages of salts; likewise with those of our study (48).

### Microbiological Analysis

No significant differences were observed in the counts of *Salmonella* spp., *Shigella, Staphylococcus*, and *L. monocytogene*s (data not shown) and complied with the limits established by Regulation (EC) 2073/2005, relative to the microbiological criteria applicable to food products.

Slightly acidified fermented sausages often have a pH > 5.3, so one of the safety barriers, acidity, is lost, thus facilitating the development of certain pathogenic microorganisms. In this study, the pH values (Table 3) were kept below what was established, so it was not considered a risk factor for microbiological growth. During the chorizo fermentation process, the Enterobacteriaceae counts (*Salmonella*) remained constant. Throughout curing, they did not develop differences in growth (CFU/g) for this microorganism, obtaining an absence in 25 g of chorizo. This result may be because not only NaCl is a total barrier to microbial growth, but it may also influence the addition of Na nitrites in the formulation, since this ingredient can slow bacterial growth (12). Ibáñez et al. obtained similar results, they did not observe significant differences in the analyses conducted on the control sample and on the modified sample (partial substitution of KCl), in the count of *Salmonella* spp., *Staphylococcus aureus, Clostridium* sulphite-reducing and *Escherichia coli* (49). In sausages, *S. aureus* is found in low levels (50). NaCl and nitrites have little effect on this microorganism, but under acidic pH and low temperatures its growth is considerably hampered. Another determining factor in the growth of this microorganism is the a_w_, as it is vitally important this is kept below 0.955 (51). In the specific case of this study, the a_w_ of all the chorizo formulation was below 0.9 (Table 3).

### Sensory Analysis of the Iberian Chorizo at the End of Its Curing

The results of the sensory analysis of chorizo are shown in Table 6. Significant differences are observed in all the sensory attributes studied (*P* < 0.05), except in the odor (*P* > 0.05). The CTRL sample was the worst valued by consumers. This may be because the composition of the food and the release of its aroma affects the release of salivary compounds, the most implicated being: mucins, some enzymes and small molecules such as salts (52). High salivary salts increase the release of flavorings in the oral cavity due to the salty effect, resulting in abnormal and undesirable flavors (53).

This negative assessment, regarding appearance, may be because the CTRL chorizo developed an unwanted colouration. The L^*^ value in the instrumental measure of color (Table 6) showed the CTRL chorizo developed a whitish colouration compared to KCl and SC. Therefore, the consumer preferred chorizos with a darker color. The salty flavor was better valued in the SC chorizo than in the CTRL, thus using other salts did not affect the acceptance of salty.

Cevallos observed a lower hardness and worse texture in pork ham with a partial substitution of NaCl for KCl. However, in this study, the SC chorizos obtained a better texture rating than CTRL and KCl, not observing significant differences between the latter two and agreeing with what was observed in cured loin (17). The texture of the food has a great influence on the chewing process, in particular on the number of chewing cycles, as a hard, dry food requires a greater number of cycles to be fragmented into particles and impregnated by saliva before being swallowed (54). Another study by Horita et al. found no differences in appearance and aroma in “mortadela” made with different chloride salts (32). Regarding the overall flavor and acceptability of the product, the SC chorizo was the best valued by consumers, with the KCl chorizo being the second best valued and then the CTRL. The substitution of salt for other salts has improved its sensory properties, observing significant differences in the SC chorizos, in most attributes studied, compared to the CTRL and KCl. During the oral processing of a food, sensory characteristics are developed, some of them evaluated in this study, due to the release of compounds responsible for them, and which have been previously mentioned. This leads to the perception of the organoleptic properties of the food, contributing significantly to the acceptance of the product by consumers (55), being in this research the SC chorizo the best valued by the consumer panel.

## Conclusions

The partial replacement of NaCl using a mixture of salts (KCl and SC) has a great influence on the moisture content of Iberian chorizo, since the use of salts hinders water loss from the food composition. Using a combination of mineral salts, from seawater, could be considered an alternative in the complete substitution of other chloride salts, since its use has reduced the Na content by up to 30.15%. Using the combined salts (SC) considerably reduces the concentration of salt in the final product, allowing nutritional and health claims for most minerals studied in its labeling. In addition, using low salt content favors the activity of proteases, and increases the proteolytic activity of sausages, thus favoring their sensory characteristics. However, this reduction of NaCl showed a loss of the reddish hue typical of this sausage, making it darker. The partial substitution of NaCl did not negatively affect the sensory characteristics, but also with SC chorizos, they were better valued by the consumer panel.

Finally, using NaCl substitutes does not compromise the stability and safety of the final product, as they meet the required microbiological conditions established in their corresponding regulation. The reformulation conducted in the SC and KCl chorizos give Na values that result in a product that could carry the nutritional claim “reduced sodium/salt content” compared to a similar product. Therefore, it is possible to produce a cured sausage with reduced Na content, by partially substituting it with KCl, or by replacing it with a combination of mineral salts.

## Data Availability Statement

The original contributions presented in the study are included in the article/supplementary material, further inquiries can be directed to the corresponding author.

## Author Contributions

LT, ES, and AA: conceptualization. EÁ, BM, and ES: methodology. LT, AA, BM, and ES: investigation. LT and LB-M: data curation. LB-M, LT, and AP: writing-original draft preparation. LT, LB-M, and AP: writing-review and editing. All authors: have read and agree to the published version of the manuscript.

## Conflict of Interest

The authors declare that the research was conducted in the absence of any commercial or financial relationships that could be construed as a potential conflict of interest.
